# L-Theanine Reduced the Development of Knee Osteoarthritis in Rats via Its Anti-Inflammation and Anti-Matrix Degradation Actions: In Vivo and In Vitro Study

**DOI:** 10.3390/nu12071988

**Published:** 2020-07-03

**Authors:** Hui Bai, Zhiheng Zhang, Yue Li, Xiaopeng Song, Tianwen Ma, Chunpeng Liu, Lin Liu, Rui Yuan, Xinyu Wang, Li Gao

**Affiliations:** 1 Heilongjiang Key Laboratory for Laboratory Animals and Comparative Medicine, College of Veterinary Medicine, Northeast Agricultural University, Harbin, Heilongjiang 150030, China; baihui101214@163.com (H.B.); zzh449756020@163.com (Z.Z.); 2 College of Veterinary Medicine, Northeast Agricultural University, Harbin, Heilongjiang 150030, China; liyue_315207218@163.com (Y.L.); daodao2019@163.com (X.S.); dnmatianwen@163.com (T.M.); liuxiaolin0413@163.com (L.L.); yr1074732045@163.com (R.Y.); wxy19960210@163.com (X.W.); 3 College of Animal Science and Technology, Zhongkai University of Agriculture and Engineering, Guangzhou, 510225, China; liuchunpeng@163.com

**Keywords:** OA, L-theanine, rat, anti-inflammation, anti-matrix degradation

## Abstract

The etiology of osteoarthritis (OA) is multifactorial, with no effective disease-modifying-drugs. L-theanine has been reported to inhibit inflammatory responses in some diseases and this study aimed to investigate the effect of L-theanine on Interleukin-1(IL-1)β-stimulated chondrocytes, and in an injury-induced OA rat model. Primary chondrocytes were stimulated by IL-1β (10 ng/mL) for 24 h and then co-cultured with L-theanine for 24 h. The effects of L-theanine on IL-1β-stimulated expression of pro-inflammatory cytokines and hydrolytic enzyme were analyzed using Western blotting, quantitative polymerase chain reaction (q-PCR) and enzyme-linked immunosorbent assay (ELISA) kits. An immunofluorescence assay was used to detect nuclear factor kappa B (NF-κB) phosphorylation. OA was induced by anterior cruciate ligament transection (ACLT) surgery in rats and celecoxib was used as a positive control. OA severity was measured using the Osteoarthritis Research Society International (OARSI) grading system to describe histological changes. The results showed that L-theanine decreased the expression of pro-inflammatory mediators, including cyclooxygenase-2 (COX-2), prostaglandin E2 (PGE-2), inducible nitric oxide synthase (iNOS), and nitric oxide (NO), both in vivo and in vitro. L-theanine treatment inhibited IL-1β-induced upregulation of matrix metalloproteinases (MMP)-3 and MMP-13, as well as inhibited NF-κB p65 activation. In vivo animal model showed that L-theanine administration (200 mg/kg) significantly alleviated OA lesions and decreased OARSI score. Our data indicated that L-theanine decreased inflammatory cytokines and protected extracellular matrix degradation through inhibition of the NF-κB pathway, and L-theanine may be considered a promising therapeutic strategy in OA prevention.

## 1. Introduction

Low-grade inflammation plays an important role in osteoarthritis (OA) [1,2], and pre-clinical and clinical trials support therapeutic targets for inflammation in OA treatment. Inflammation cytokines such as cyclooxygenase-2 (COX-2) and prostaglandin E2 (PGE-2) that cause an increase in OA pain with OA progression, and non-steroidal anti-inflammatory drugs (NSAIDs) can selectively inhibit COX-2, and serve as effective drugs in OA management [3]. IL-1β is a pro-inflammatory factor found in synovial fluid and cartilage during OA development, and induces the production of catabolic mediators [4], such as matrix metalloproteinases (MMPs), which degrade proteoglycans and type II collagen, and is a component of extracellular matrix (ECM) scaffolds [5]. MMP-3 and MMP-13 have been reported as two key mediators in joint inflammation and degradation [6,7]. The dysfunction and metabolic disorders of chondrocytes and ECM may cause pathological changes to articular cartilage [8]. These factors are locally secreted by chondrocytes, synovial cells, and macrophagocytes, and aggravate cartilage damage through receptors on the cell surface and crosstalk between signaling pathway. Importantly, nuclear factor kappa B (NF-κB) was reported a key promoter in OA inflammation [9,10], which is induced by pro-inflammatory mediators, and in turn promotes cytokine production, including IL-1, IL-6, and tumor necrosis factor-α (TNF-α) [11]. In addition, NF-κB is essential for the production of catabolic proteins, including MMPs and iNOS.

Currently, drugs that can effectively slow down the degradation of OA cartilage are still under investigation. The most commonly used medications, including NSAIDs, such as ibuprofen and celecoxib have side-effects involving gastrointestinal ulcer bleeding during long-term administration, and these drugs are not appropriate for patients with kidney damage [12]. Some polyphenols are used to prevent the progression and development of OA [13,14,15], such as curcumin, epigallocatechin gallate (EGCG), and resveratrol. However, more clinical trials are needed to confirm the effects of these drugs, and identifying new disease-modifying drugs is of utmost importance.

L-theanine is the main amino acid in green tea and accounts for more than 50% of total free amino acids [16,17]. Other constituents of green tea, for example, EGCG, have been reported to have OA protective effects in human chondrocytes [18] and in a post-traumatic OA mouse model [19]. Biological activities of L-theanine, including anti-inflammatory, antioxidant, anti-tumor, neuroprotective effects, immune regulation [20,21,22,23], and inhibiting the NF-κB pathway have been observed. However, the role of L-theanine in OA inflammation and whether it can regulate the metabolic activity of chondrocytes are still unclear.

Based on this, we proposed a hypothesis that L-theanine plays a protective role in IL-1β-induced OA chondrocytes for rats in vitro, and alleviates cartilage injury in an OA model of anterior cruciate ligament transection (ACLT) in vivo. Celecoxib was chosen as a positive control for treatment because of its powerful anti-inflammatory activity and fewer side effects [3] compared with other NSAIDs.

## 2. Materials and Methods

### 2.1. Preparation of Rat Primary Chondrocytes

Primary chondrocytes were isolated from 14- to 21-day-old rats. After trypsinization (0.25%, GIBCO, New York, USA) of cartilage for 30 min, the slice was treated with 0.2% collagenase II for 4 h at 37 °C. The culture medium was then cultured with DMEM/F12, including 10% fetal calf serum (Biological Industries, Israel). The steps were consistent with previous research [24] and the second passage cells were chosen for the subsequent experiments. Identification of chondrocytes was stained with toluidine blue for proteoglycans and immunohistochemical staining for type II collagen (Novus Biologicals, CO, USA).

The medium was cultured with DMEM/F12 containing 0.5% serum starving for 12 h and were stimulated with IL-1β (PeproTech Inc. USA) for 24 h prior to being co-cultured with different concentrations of L-theanine (50, 100, 200 μM) for 24 h. Cells treated with IL-1β only served as the control. The dose of L-theanine (≥98% (high-performance liquid chromatography, HPLC), Sigma-Aldrich, St. Louis, MO, USA) was determined according to previous studies which L-theanine showed inhibition of NF-κB [25] and anti-inflammation activities [26].

### 2.2. Cell Proliferation Assay

Chondrocytes were inoculated into 96-well plates (5000/well) for 24 h. The culture medium was administrated with or without IL-1β for 24 h and different concentrations of L-theanine (0, 10, 50, 100, 200 400, and 800 μM) treated for 24 h, and then incubated with cell counting kit (CCK)-8 solution (DOJINDO, Japan) for 1 h at 37 °C, which was used to determine whether L-theanine had cytotoxic effects, and to identify the effective dose of L-theanine for the chondrocytes stimulated by IL-1β. Cells were cultured in DMEM containing 0.5% serum for 12 h prior to L-theanine administration to ensure that cells were quiescent.

### 2.3. In Vitro Immunofluorescence Assay

The culture medium was rinsed with phosphate-buffered saline (PBS) prior to 4% paraformaldehyde fixation for 1 h at ambient temperature (AT). Chondrocytes were permeabilized with PBS containing 0.2% Triton-X100 for 30 min at AT before incubation with primary antibodies (phospho-p65 from Cell Signaling Technology, USA, 1:100 in PBS) in a wet box (4 °C overnight). The secondary antibody (goat-anti-rabbit immunoglobulin, diluted 1:250 in PBS) was used to treat the glass plate for 1 h and subsequently incubated with 4’,6-diamidino-2-phenylindole (DAPI) (Beyotime Biotechnology, Shanghai, China) for 3 min and Phalloidin (Alexa Fluor^®^ 555) for 15 min at AT. The processing interval was rinsed gently with PBS containing Tween-20 (PBST). The medium was visualized under fluorescence microscope.

### 2.4. In Vitro Western Blot Analysis

Western blot analysis was performed as described in our early study [27] to detect the expression of both nucleoproteins and cytoplasmic proteins for p-65 and p-p65 (Beyotime Biotechnology, Co., Ltd., Shanghai, China). Total protein was extracted to measure the contents of MMP-3 (Cell Signaling Technology, Inc, Boston, MA, USA), MMP-13 (Novus Biologicals, Inc, CO, USA), COX-2 (Cell Signaling Technology, Inc, Boston, MA, USA) and iNOS (Novus Biologicals, Inc, Littleton, CO, USA). An enhanced BCA protein assay kit (Beyotime Biotechnology, Co., Ltd., Shanghai, China) was used to detect the concentration of protein.

### 2.5. In Vitro Real-Time Polymerase Chain Reaction (PCR) Analysis

Total RNA was extracted from the cultured chondrocyte monolayers through the RNAiso Plus reagent, according to the manufacturer’s instructions. cDNA was obtained through a reverse transcriptase kit (TianGen Biotechnology, Beijing, Co., Ltd. China) with gDNA remover. Real-time quantitative PCR was performed in duplicate to determine the relative gene expression of MMP-3 and MMP-13, with an endogenous control of glyceraldehyde-3phosphate dehydrogenase (GAPDH). Primer sequences (Sangon Biotech, Shanghai, Co., Ltd., China) are provided in Table 1.

### 2.6. In Vivo Rat Anterior Cruciate Ligament (ACL) Transection-Induced Osteoarthritis (OA) Model

Ten to 11-week-old male Sprague Dawley (SD) rats with an average weight range from 230 g to 270 g (bought from Harbin Medical University, Harbin, China) were used to establish an experimental model by anterior cruciate ligament transection (ACLT). Rats were raised at the experimental animal center in Northeast Agricultural University on a standard 12 h dark/light cycle. All animal experiments were carried out in accordance with the guidelines of the China Ethical Committee for Animal Experiments.

**Figure 1:**
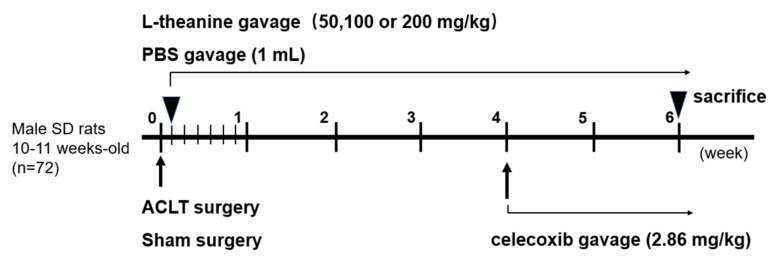
The intervention time point in vivo; osteoarthritis (OA) was induced in 10- to 11-week-old male Sprague Dawley (SD) rats by anterior cruciate ligament transection (ACLT) of the right knee. Sham surgery was performed on the right knee by incision of joint capsule, and sham operation was performed from a separated group of rats. L-theanine (50, 100 or 200 mg/kg, once a day gavage, until the rats were sacrificed), was administrated one day after ACLT surgery. Phosphate-buffered saline (PBS, 1 mL) was used for treatment after sham surgery as a control. Celecoxib (2.86 mg/kg) was used for treatment 4 weeks after ACLT surgery as a positive control.

A total of 72 rats were anesthetized (about 3.5% isoflurane, obtained from Shenzhen Ruiwode Life Technology Co., Ltd., Shenzhen, China) and the joint capsule incision and ACLTs were performed under an operating microscope. Rats were randomly divided into 6 groups: (1) ACLT group (no treatment), (2) Sham group (capsule incision only, PBS treatment), (3) Low-dose L-theanine (50 mg/kg), (4) Moderate-dose L-theanine (100 mg/kg), (5) High-dose L-theanine (200 mg/kg), (6) Celecoxib group (2.86 mg/kg [28]). The drug delivery route and dosage are presented in Figure 1. L-theanine was bought from Shanghai yuanye Bio-Technology Co., Ltd. (BR, 99%).

### 2.7. Histological Assessment and Osteoarthritis Research Society International (OARSI) Grading System

The tibial samples of the right knee were collected for decalcification prior to a standard paraffin embedding. The block was sectioned at 5 µm-thick slices, separated by 250 μm. Sections were stained with hematoxylin-eosin (HE) to assess the changes of cartilage and Safranin O and fast green for proteoglycan degeneration. OA severity was evaluated blinded using the OARSI grading system, which was suitable to both experienced and novice scorers [29]. Cartilage severity was displayed by OARSI scores from 0–6.

### 2.8. In Vivo Enzyme-Linked Immunosorbent Assay (ELISA) Kits

Serum samples and cartilage tissue samples were collected and concentrations of COX-2, PGE-2, iNOS and NO were measured using specific rat enzyme-linked immunosorbent assay (ELISA) kits (NanJing JianCheng, Co., Ltd., NanJing, China) according to the manufacturer’s instructions. Col2-3/4C-terminalcleavageproductoftype II collagen (C2C) and crosslinked C-telopeptides of Type II collagen (CTX-II) assay kits were bought from Jingmei Biotechnology Co., Ltd. (Jingmei, Co., Ltd., JiangSu, China).

### 2.9. Statistical Analysis

All statistical analyses were performed using SPSS 22.0 software, and results are expressed as the mean ± standard deviation (SD). One-way analysis of variance (ANOVA) was used for comparisons between groups, and P < 0.05 was considered statistically significant. Multiple comparisons between groups were performed using post hoc Student–Newman–Keuls tests. Western blot results were analyzed using Image J software. Histological analyses were performed by two experienced investigators who were double-blinded. All experiments were performed in triplicate.

## 3. Results

### 3.1. L-Theanine Reduces the Release of Catabolic Enzymes and Inflammatory Mediates from IL-1-Induced Chondrocytes In Vitro

**Figure 2:**
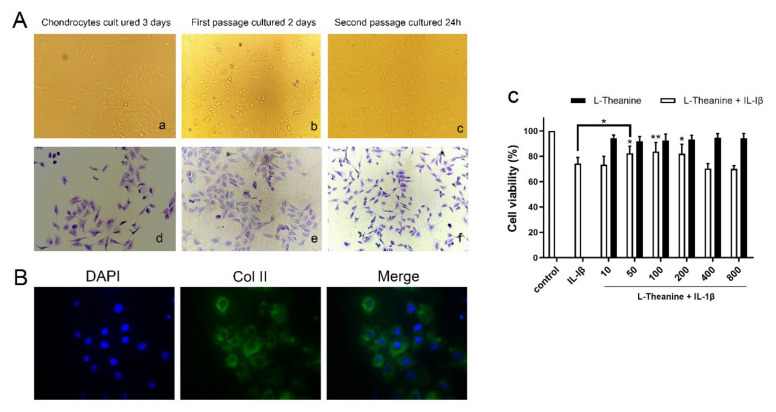
Identification of primary chondrocytes and the effects of L-theanine on cell viability with or without IL-1β. (**A**) Representative images of rat primary chondrocyte morphology, which was observed on the third day after incubated, the second day after the first passage of cells and 24 h after the second passage of cells, respectively(a-c). Representative images of toluidine blue staining of monolayers of rat chondrocytes in the same time point (d–f) and the nucleus of cells was dyed blue violet. Original magnification × 100. (**B**) Immunofluorescence assay of rat second generation chondrocytes for type II collagen with primary antibody to COL2A1 and fluorescent secondary antibody. DAPI for nucleus staining. Original magnification × 200. (**C**) CCK-8 assay for cell ability of L-theanine with or without IL-1β. L-theanine treated with different concentrations (0, 10, 50, 100, 200 400, 800 μM) showed no significant difference compared to controls. Pre-treatment with IL-1β (10 ng/mL) for 24 h sharply reduced cell viability, however the significant increase was observed after treatment of L-theanine in 50, 100, and 200 μM for 24 h. Values are the mean ± standard deviation (SD); * *p* < 0.05, ** *p* < 0.01 vs. IL-1β group.

The cultured cells showed fast proliferation and a polygonal appearance (Figure 2A). Proteoglycan were stained blue-ish violet by toluidine blue staining (Figure 2A). In addition, the immunofluorescence staining results showed that the green fluorescence of cell endochylema was type II collagen (Figure 2B). Moreover, L-theanine treatment alone resulted in no demonstrable adverse effects on cell viability, which was in accordance with the results of the CCK-8 assay. When L-theanine was co-cultured with IL-1β, the results showed a significant increase in cell viability at 50 μM of L-theanine that was sustained up to 200 μM of L-theanine (Figure 2C). Therefore, 50, 100, and 200 μM were selected for subsequent experiments.

**Figure 3:**
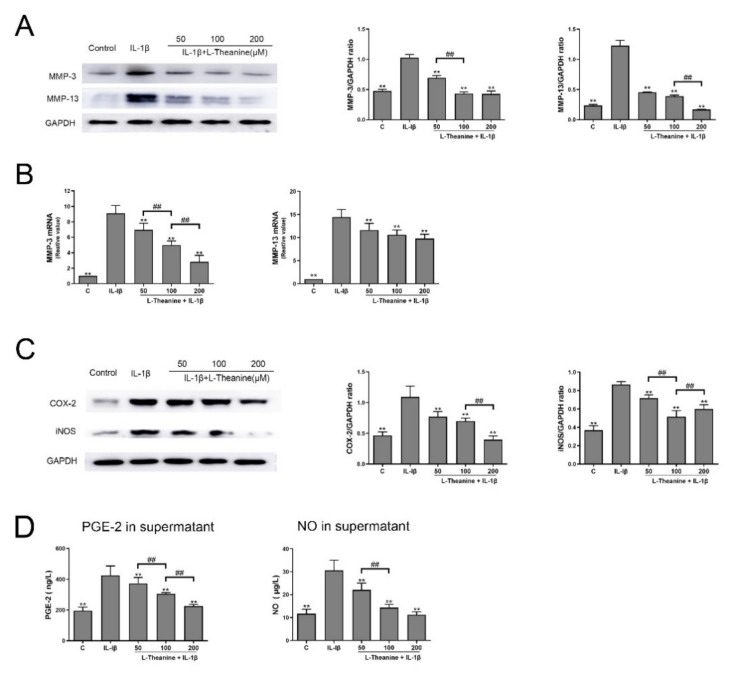
L-theanine reduced the expression matrix-degrading enzymes and pro-inflammatory mediates in IL-1β-stimulated rat chondrocytes. (**A**) Western bolt analysis of MMP-3 and MMP-13 in chondrocytes treated with L-theanine (50, 100, 200 μM) for 24 h. (**B**) Real time PCR analysis of gene expression of MMP-3 and MMP-13 in chondrocytes treated with L-theanine (50, 100, 200 μM) for 24 h. (**C**) Western bolt analysis of cyclooxygenase-2 (COX-2) and iNOS in chondrocytes treated with L-theanine (50, 100, 200 μM) for 24 h. (**D**) Enzyme-linked immunosorbent assay (ELISA) of prostaglandin E2 (PGE-2) and NO in the cell supernatant treated with L-theanine (50, 100, 200 μM) for 24 h. Values are the mean ± SD; ** *p* < 0.01, vs. IL-1β treatment group. ## *p* < 0.01 vs. L-theanine (100 μM) treatment group. C, control.

To determine the effects of L-theanine on IL-1β-induced chondrocytes, we investigated its effect on the expression of MMPs and pro-inflammatory cytokines. After 24 h of co-culturing with IL-1β, L-theanine dose-dependently decreased mRNA levels and protein levels of MMP-3 and MMP-13 (Figure 3A–B). Furthermore, L-theanine also reduced the expression of COX-2 and iNOS in chondrocytes, as well as the secretion of PGE-2 and NO in culture supernatant in a dose-dependent manner (Figure 3C–D).

### 3.2. L-Theanine Inhibits Nuclear Factor Kappa B (NF-κB) p65 Phosphorylation and Expression In Vitro

**Figure 4:**
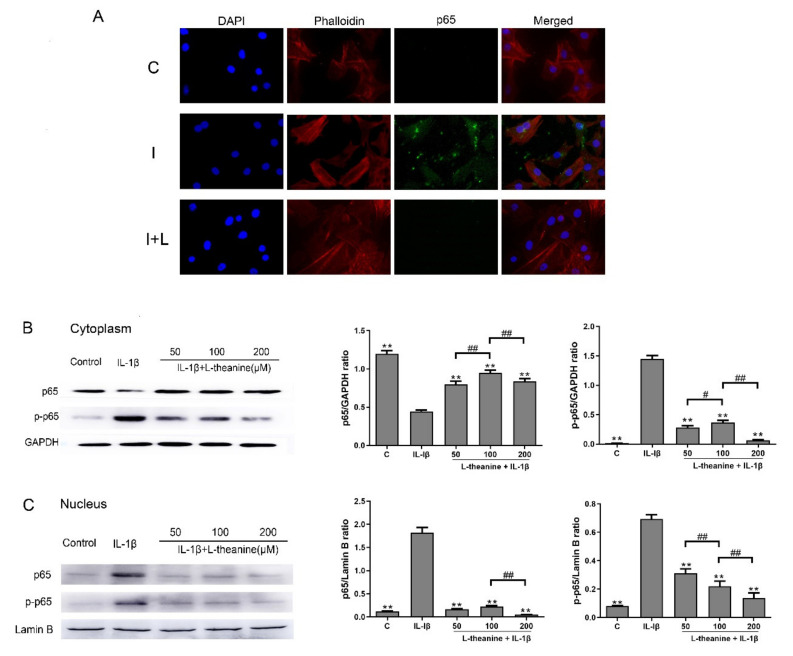
L-theanine inhibited nuclear factor kappa B (NF-κB) phosphorylation and expression in IL-1β induced chondrocytes in vitro. (**A**) Immunofluorescence analysis of nuclear translocation of NF-κB in chondrocytes. NF-κB signaling in chondrocytes was activated (green fluorescence) after IL-1β (10 ng/mL) treatment for 24 h and was suppressed after L-theanine (200 μM) treatment for 24 h. C, control; I, IL-1β (10 ng/mL); I + L, IL-1β (10 ng/mL) + L-theanine (200 μM). (**B**) Western blot analysis of p65 and p-p65 in cytoplasm in rat chondrocytes. The internal reference was GAPDH. (**C**) Western blot analysis of p65 and p-p65 in nucleus in rat chondrocytes. The internal reference was Lamin B. Values are the mean ± SD; ** *p* < 0.01 vs. IL-1β treatment group. # *p* < 0.05, ## *p* < 0.01 vs. L-theanine (100 μM) treatment group. C, control; p-p65, phosphorylated p65.

To unravel the mechanisms by which L-theanine promoted inflammatory responses and triggered ECM degradation, we next investigated, using Western blot analysis and immunofluorescence analysis, whether L-theanine inhibited the phosphorylation and expression of NF-κB p65 in chondrocytes. NF-κB is a multifunctional transcription factor associated with proinflammatory responses, and plays an important role in OA progression [30]. We found that L-theanine, given at 50 μM to 200 μM, significantly inhibited the expression of phosphorylated NF-κB p65 (Figure 4A–C). Notably, L-theanine in 200 μM exerted intense effects (Figure 4B–C).

### 3.3. L-Theanine Ameliorates Knee Joint Histopathology and Reduces Extracellular Matrix (ECM) Degradation in the Rat Anterior Cruciate Ligament Transection (ACLT) Model

**Figure 5:**
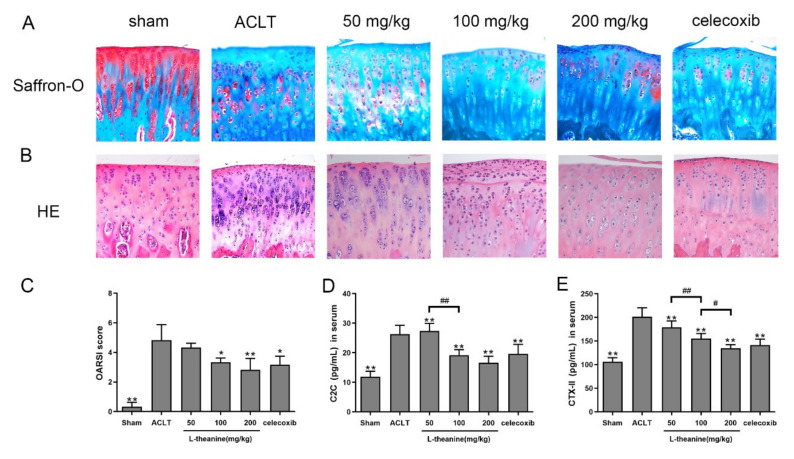
L-theanine treatment ameliorated OA cartilage lesions and reduced serum levels of cartilage metabolism biomarkers. (**A–C**) Representative images of cartilage in tibial plateau (right knees of rats) and Osteoarthritis Research Society International (OARSI) score. Safranin O-fast green staining and HE staining were performed to evaluated the relative content of proteoglycan (red color in Safranin O staining) and articular cartilage degeneration. Original magnification ×200. HE, hematoxylin-eosin staining. (**D–E**) ELISA assay of serum levels of C2C and CTX-II in vivo. Values are the mean ± SD; * *p* < 0.05, ** *p* < 0.01 vs. ACLT group. # *p* < 0.05, ## *p* < 0.01, compared between groups with L-theanine treatment.

To assess the effect of L-theanine on OA development and progression, we performed ACLT surgery in rats with or without L-theanine administration. Celecoxib was used as a positive control. Lesions of the medial tibial plateau were analyzed to assess knee joint damage, including articular cartilage degeneration, and proteoglycan content by Safranin-O staining and chondrocyte proliferation. In ACLT knees, severe cartilage degradation was observed, including moderate to severe hypocellularity, superficial fibrillation, a thickened perichondrium, and a reduction in Safranin-O staining when compared with sham-operated knees (Figure 5A). Administration of L-theanine (100 and 200 mg/kg) caused significant reduction in cartilage degradation and an increase in Safranin-O staining (Figure 5A), thereby indicating increased expression of proteoglycans. Moreover, milder superficial fibrillation and matrix edema were noted by treatment with celecoxib when compared with the ACLT rat model (Figure 5A). OA lesions were less severe with both L-theanine and celecoxib treatment when compared with the ACLT surgery group, however L-theanine at 200 mg/kg showed a similar OARSI score to celecoxib treatment as assessed by the OARSI score system (Figure 5C). Results were confirmed by HE staining. The operated knees from L-theanine-treated rats showed less severe cartilage injury, a lack of cell clustering, and clear columnarization when compared with the ACLT group (Figure 5B).

We next investigated the levels of C2C and CTX-II, two biochemical markers that are potentially predictive of the development of knee OA. A significant increase in serum levels of C2C and CTX-II were observed at 6 weeks after ACLT surgery in rats (Figure 5D–E). After L-theanine treatment, both of these two degradation products of type collagen II were significantly decreased in a dose-dependent manner (Figure 5D–E).

### 3.4. Systemic L-Theanine Treatment Exerts Anti-Inflammatory Activity In Vivo

**Figure 6:**
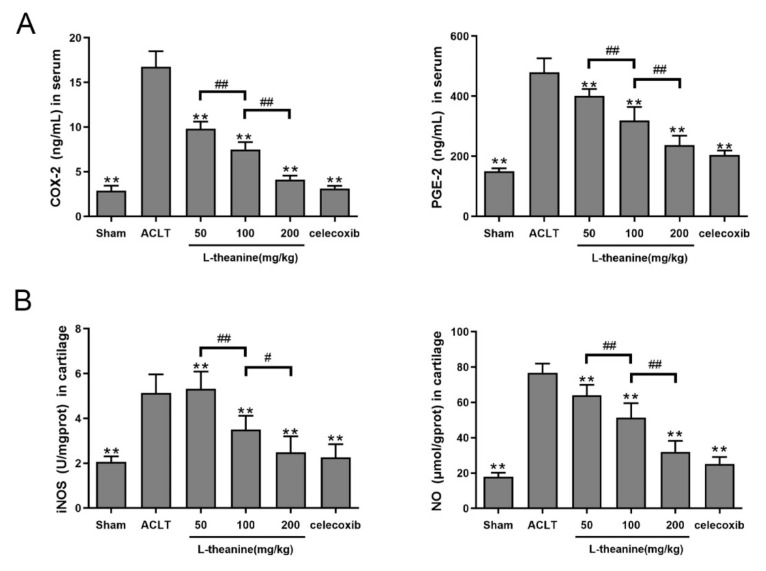
L-theanine reduced levels of pro-inflammatory mediates in vivo. (**A**) ELISA assay of serum levels of COX-2 and PGE-2 in ACLT-induced OA rat model treated with L-theanine (50, 100, 200 mg/kg) and celecoxib. (**B**) ELISA assay of iNOS and NO in cartilage in ACLT-induced OA rat model. Values are the mean ± SD; ** *p* < 0.01 vs. ACLT group. # *p* < 0.05, ## *p* < 0.01, compared between groups with L-theanine treatment.

To investigate whether L-theanine could still inhibit inflammatory responses in a surgery-induced OA model, ELISA assays were performed. The results showed that significantly increased serum levels of COX-2 and PGE-2, and highly increased levels of iNOS and NO were observed in rats that underwent ACLT surgery without L-theanine treatment. Administration of L-theanine (3 different doses), commencing 1 day after ACLTsurgery (lasting 6 weeks of treatment), could significantly decreased the levels of these pro-inflammatory mediators in a dose-dependent manner. Of note, L-theanine at 200 mg/kg had a similar effect when compared with celecoxib treatment (Figure 6A–B).

## 4. Discussion

This study was the first to demonstrate the effects of L-theanine on articular cartilage in experimentally induced OA and IL-1β-induced chondrocytes. The present study was designed based on previous reports that showed that cartilage and synovial inflammation occurs in OA progression [10,31,32], and that L-theanine can prevent inflammatory responses by suppressing the NF-κB signaling pathway [33,34,35], and reduces the release of downstream pro-inflammatory mediators [26,36] in inflammatory-related diseases.

Animal models of OA which was induced by surgery was demonstrated mimic human post-traumatic OA, including partial or total meniscectomy, destabilization of the medial meniscus (DMM), anterior cruciate ligament (ACL) or posterior cruciate ligament transection. Rat OA model was broadly used and disease progression in rat surgical models is much faster than in human OA [26]. Except for rapid progression and low cost, a rat ACLT induced OA model exhibits mild cartilage destruction 4 weeks post-surgery. These advantages make rat OA models, especially ACLT, suitable for drug testing [37].

In our study, we used 3 different doses of L-theanine, 50, 100, and 200 mg/kg respectively. We selected these doses because in a previous study it was demonstrated that L-theanine (50 mg/kg/day, p.o.) exerted neuroprotective effects by inhibiting the NO production [38]. Moreover, the antioxidant effect of L-theanine (50 mg/kg/day, p.o.) on ethanol-induced oxidative stress was demonstrated by inhibiting lipid peroxidation in mice [33]. Considering that the concentration of oral drugs may be diluted after reaching the joints, we examined a higher drug concentration to evaluate the effect of L-theanine on OA. The results showed that a concentration greater than/equal to 50 mg/kg was effective for OA development, and that a concentration of 200 mg/kg showed better results. Of note, L-theanine treatment at 200 mg/kg showed similar effects when compared to treatment with celecoxib.

In this study, L-theanine significantly decreased the expression of COX-2, PGE-2, MMP-3, and MMP-13 in vitro, which are major pro-inflammatory cytokines and matrix-degrading enzymes. Moreover, systemic treatment with L-theanine had a significant ameliorating effect on the cartilage injury of OA histopathology, and reduced the levels of COX-2 and PGE-2 in vivo. Furthermore, major biomarkers of cartilage destruction were inhibited by L-theanine administration in vivo, including C2C and CTX-II. Taken together, our results indicated that application of L-theanine yields protective effects that might result from the inhibition of pro-inflammatory pathways and matrix hydrolase.

The pathogenesis of OA following ACLT surgery is multifaceted and has focused on overloaded mechanical stress to articular cartilage and inflammation of the synovium [37,39]. Mechanical compression and low-grade inflammation, which have been reported key promoters to cartilage degeneration [1,40], triggers the release of matrix hydrolase and cytokines. These catabolic mediates are considered central causes of OA cartilage deterioration [41]. COX-2 is a key enzyme in the initial synthesis process of PGE-2, and overexpression of COX-2 has resulted in elevated expression of PGE-2 [42]. Several studies have reported that COX-2 and PGE-2 were upregulated in OA and promoted cartilage damage through activating inflammatory pathways [43,44]. COX-2 specific inhibitors, including celecoxib, are representative drugs of NSAIDs and have been successfully used as an alternative for OA treatment. In our study, celecoxib was selected as a positive control because celecoxib has demonstrated fewer side effects compared with non-specific COX inhibitors [45,46]. The data presented in the current study showed that levels of COX-2 and PGE-2 were downregulated after celecoxib administration in vivo, however, histological assessment showed no significant changes in proteoglycans (positive staining of cationic with Safranin O). It was unlikely that COX-2 inhibition alone could reduce degradation of proteoglycans and prevent OA cartilage damage in in vitro chondrocyte explants [45]. In addition, our results showed that L-theanine treatment reduced levels of COX-2 and PGE-2 both in vivo and in vitro, thereby indicating that L-theanine could suppress inflammatory responses in OA progression.

In the current study, the effects of L-theanine on iNOS and NO were investigated. Recent studies have shown the contribution of NO in OA pathogenesis [47,48]. Both iNOS and NO were significantly increased in OA development and were accompanied with up-regulation of MMPs, which contributed to collagen-destruction and proteoglycan-degradation. IL-1β and TNF-α were shown to be promoters of NO production, and NO could promote apoptosis of chondrocytes [49]. Moreover, IL-1β and NO induced the onset of matrix-degrading enzymes, including MMP-3 and MMP-13 [50]. NOS exists in animals as neuronal NOS (nNOS), endothelial NOS (eNOS), and inducible NOS (iNOS) [51]. eNOS and nNOS exert effects through combining with each other, and are in general targeted at the cardiovascular and nervous system as second-messenger molecules after being induced to NO [52]. iNOS is the enzyme that is responsible for the production of NO. The induction of iNOS expression is regulated by NF-Κβ [53] and IL-1β upregulation of the expression of iNOS is mediated by NF-kB. Thus, IL-1β results in the formation and translocation of NF-kB into the nucleus, where it binds to specific sequences in the promoter region of the iNOS gene [54].

NF-κB is a multifunctional transcription factor that is associated with proinflammatory responses [55] and regulates the expression of genes that regulate cell proliferation, differentiation, and apoptosis [56]. In a variety of tissues, the transcriptional activity of NF-κB is increased with aging, and is associated with numerous age-related degenerative diseases, including osteoporosis and OA [57]. NF-κB activation occurs in response to stimuli, including IL-1β, TNF-α, and lipopolysaccharide (LPS). OA chondrocytes display increased expression of IL-1β, which significantly promotes the catabolic metabolism of joints and cartilage through activation of the NF-κB pathway, thus inducing a variety of genes in chondrocytes, including cytokines, MMPs, and a disintegrin and metalloproteinase with thrombospondin motifs (ADAMTS) [58]. In the current study, treatment with L-theanine resulted in inhibition of the NF-κB pathway by suppression of the translocation of phosphate p-65. Our results also showed that administration of L-theanine significantly reduced the expression of iNOS and NO both in vivo and in vitro. Based on results presented in a previous study, L-theanine might prevent cartilage deterioration by reducing the action of IL-1β, NF-κB signaling, MMPs, and NO. Considering that most signaling including MAPK, Wnt, and Nrf2 pathways also play important roles in the OA process, an NF-κB inhibitor would be necessary in future studies of L-theanine.

In OA, the destruction of the articular cartilage will result in the loss of its two major components, proteoglycans and type II collagen [59], and C2C and CTX-II are two biochemical markers of the breakdown of type II collagen [60]. In our study, serum levels of C2C and CTX-II were measured to evaluate cartilage metabolism. Our results showed a reduction in C2C and CTX-II were observed in an experimentally induced rat model. The serum or urine concentration of C2C and CTX-II is sensitive [61] and can be obtained relatively easily compared to synovial fluid. However, considering that the serum concentration does not directly reflect the local environment of the joint, the effect of L-theanine on C2C and CTX-II in synovial fluid should be investigated in future studies.

Certain limitations to the current study must be acknowledged. This study was based on a rat model that underwent 6 weeks of treatment, and in the future, long-term treatment is necessary before clinical applications are performed in humans. Furthermore, the use of behavioral assessment could help evaluate OA pain following L-theanine treatment. In addition, although L-theanine exerted protective effects to cartilage lesions in the current study, considering the anatomy of the knee joint, further studies on the effects of L-theanine on subchondral bone and synovium are warranted. Although L-theanine performed protective effects on OA rat knees and rat primary chondrocytes, more tests are needed before the application for human OA treatment or prevention.

## 5. Conclusions

Our data revealed that L-theanine exerts an anti-inflammation and ECM-protection effect, and suppresses the NF-κB pathway in IL-1β stimulated chondrocytes. In addition, our in vivo study demonstrated a positive effect of L-theanine for damaged-cartilage and showed a comparable treatment effect of pro-inflammatory cytokines to celecoxib after ACLT-induced OA. These results suggest that L-theanine should be considered in OA prevention.

## Figures and Tables

**Table 1:** Primer sequences.

Gene	Primer Sequence	Product Length (bp)
MMP-3	F: TTTGGCCGTCTCTTCCATCC	175
	R: GCATCGATCTTCTGGACGGT	
MMP13	F: TTCTGGTCTTCTGGCACACG	92
	R: TGGAGCTGCTTGTCCAGGT	
GAPDH	F: GATGCCCCCATGTTTGTGAT	150
	R: GGCATGGACTGTGGTCATGAG	

## Data Availability

All data generated or analyzed during this study are included in this published article.

## Declarations

Ethics Approval and Consent to Participate the ethical treatment of animals in this study was approved by the Animal Welfare Committee protocol (#NEAU-2017-07-0360-13) at Northeast Agricultural University (Harbin, China). All efforts were made to minimize animals suffering and to reduce the number of animals used.

## Abbreviations

OA: osteoarthritis; ACLT: anterior cruciate ligament transection; Osteoarthritis Research Society International (OARSI); IL-1: interleukin-1; COX-2: cyclooxygenase-2; PGE-2: prostaglandin E2; iNOS: inducible nitric oxide synthase; NO: nitric oxide; ECM: extracellular matrix; GAG: glycosaminoglycans; MMPs: matrix metalloproteinases; NF-κB: nuclear factor kappa-B; NSAIDs: non-steroidal anti-inflammatory drugs; TNF-α: tumor necrosis factor-α; ELISA: enzyme-linked immunosorbent assay; C2C: Col2-3/4C-terminalcleavageproductoftype II collagen; CTX-II: crosslinked C-telopeptides of Type II collagen; ANOVA: analysis of variance.
